# Response to 5-fluorouracil in metastatic extramammary Paget disease of the scrotum presenting as pancytopenia and back pain

**DOI:** 10.3747/co.v16i5.374

**Published:** 2009-09

**Authors:** K.M. Beleznay, M.A. Levesque, S. Gill

**Affiliations:** * Faculty of Medicine, University of British Columbia, Vancouver, BC; † Medical Oncology, BC Cancer Agency, Vancouver, BC

**Keywords:** Paget disease, 5-fluorouracil, extramammary, metastatic

## Abstract

Extramammary Paget disease is a rare intraepithelial neoplasm of the vulvar, penoscrotal, or perianal skin. No effective therapies for metastatic disease have been reported, and prognosis for metastatic disease is poor.

Here, we report the case of an Asian man who was initially diagnosed with extramammary Paget disease of the scrotum. Three years later, the patient presented to hospital with pancytopenia and back pain. After an extensive work-up, biopsies of liver and bone marrow revealed adenocarcinoma with signet cells and immunohistochemical staining positive for keratin 7, carcinoembryonic antigen, and prolactininduced protein, but negative for keratin 20, S100, and prostate markers, consistent with his previous biopsyproven Paget disease of the scrotum. The patient was treated with 5-fluorouracil–based therapy in addition to palliative radiotherapy to selected spine levels. A promising palliative response was demonstrated following 5-fluorouracil chemotherapy.

A review of the literature on the pathogenesis, diagnosis, treatment options, and outcomes for metastatic extramammary Paget disease is presented.

## 1. CASE REPORT

A 67-year-old Asian man presented to a community hospital after several weeks of severe fatigue and back pain requiring opioid analgesics. He denied fevers, night sweats, weight loss, gastrointestinal bleeding, and obstructive urinary symptoms. He had a prior history of a remote right middle cerebral artery territory stroke with residual hemiparesis, diabetes, hypertension, hypercholesterolemia, and 60 pack– years of cigarette smoking. Three years earlier, he had been diagnosed with biopsy-proven extramammary Paget disease of the scrotum. He initially failed to respond to topical steroids, and he declined Mohs micrographic surgery. He was therefore treated with imiquimod for 1 year with complete resolution on re-biopsy. Since that time, colonoscopy had been performed with no abnormalities identified, and he had been well in the interim. His family history was negative for malignancy.

At presentation, this patient’s physical examination was remarkable only for left-sided pyramidal neurologic findings, hepatomegaly, and a diffusely enlarged prostate. Initial lab work found pancytopenia with leukoerythroblastic features, and repeated red blood cell and platelet transfusions were subsequently required. Serum creatinine, calcium, and liver function tests were within normal limits. Computed tomography (ct) imaging showed moderate left hilar and para-aortic lymphadenopathy, multiple liver lesions, bi-lobar prostate enlargement, and left hydronephrosis secondary to ureteral obstruction. A left ureteral stent was placed.

**FIGURE 1:**
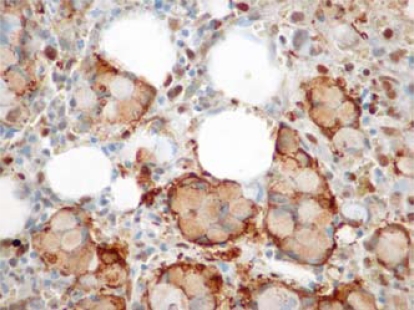
Bone marrow biopsy.

**FIGURE 2:**
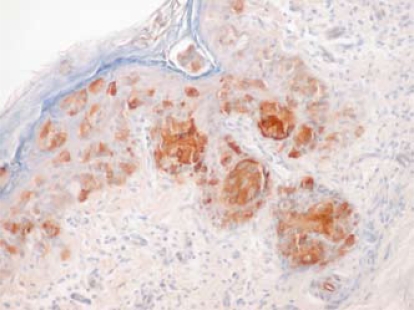
Scrotal skin biopsy.

A transrectal prostate biopsy showed Gleason 3+4 adenocarcinoma in 3 of 8 core specimens without perineural or extraprostatic extension. Prostatespecific antigen was 17.7 ng/mL. In contrast with the prostate biopsy, bone marrow aspiration and biopsy demonstrated marrow packed with poorly differentiated adenocarcinoma with signet ring cells showing positive immunohistochemical staining for keratin 7 (krt7) and prolactin-induced protein (pip: Figure 1), and also carcinoembryonic antigen (cea), but without staining for prostate-specific antigen, prostate-specific acid phosphatase, P504S, CD45, keratin 20 (krt20), vimentin, TTF1, CDX2, S100, or estrogen receptors. Fine-needle aspiration biopsy of a liver lesion was also performed and showed adenocarcinoma cells with signet ring morphology, again unlike the prostate findings. Re-examination of a previous scrotal skin biopsy confirmed the presence of extramammary Paget disease, but also showed the presence of signet ring cells with krt7, cea, and pip immunostaining (Figure 2) identical to that seen in the bone marrow and liver biopsies. Periodic acid Schiff and mucicarmine staining were also present. Dermal invasion by Paget cells was not evident, but could not be ruled out because of the superficial nature of the biopsy. Serum cea was elevated at 24 μg/L, but alfa fetoprotein and cancer antigen 19–9 were normal at 1.5 μg/L and <2×10^3^ U/L respectively.

On repeat ct scan, lesser sac and celiac lymphadenopathy were now present, as were lytic lesions in the pelvis and in all vertebral bodies, with compression fractures at sites of pain at T5, T8, and L1. A bone scan using ^99^Tc revealed widespread skeletal metastases, including vertebral involvement at multiple levels.

The patient received palliative radiotherapy in a single fraction of 800 cGy using a direct posterior field. Given the signet ring differentiation on the bone marrow and liver biopsies, an occult upper gastrointestinal malignancy was suspected, and a gastroduodenoscopy was performed, which was normal. Metastatic extramammary Paget disease remained the lead diagnostic possibility on the differential.

Despite a poor performance status, the patient received a single cycle of dose-reduced bolus 5-fluorouracil (5fu: 325 mg/m_2_ daily for 5 days) and leucovorin (20 mg/m_2_ daily for 5 days) with intent to assess treatment tolerance and response. Within 1 week, the patient’s need for red blood cell transfusions every other day ceased, and his hemoglobin remained stable for the next 2 months. His energy level and appetite improved, his weight stabilized, and his analgesic use markedly diminished with an improvement of his Eastern Cooperative Oncology Group performance status to 1. Following a second cycle of bolus 5fu and leucovorin chemotherapy, ct imaging showed improvement in the size and appearance of the liver lesions; however, skeletal disease was unchanged. The level of cea declined to 16 μg/L from 24 μg/L.

The patient received a third cycle of 5fu and leucovorin before being switched to oral capecitabine 1000 mg/m_2_ twice daily for 14 days, repeated every 21 days. He completed three such cycles with minimal side effects, but became increasingly fatigued, jaundiced, and confused before the fourth cycle. Red blood cell transfusions were again needed, and cea increased to 67 μg/L.

After a 20-week period of disease control on therapy, ct imaging showed progression of the liver disease with new ascites and peritoneal carcinomatosis. Active treatment was discontinued, and the patient was transferred to a hospice, where he died 6.2 months from the time of presentation. An autopsy was not performed.

## 2. DISCUSSION

Extramammary Paget disease is a rare intraepithelial neoplasm of the vulvar, penoscrotal, or perianal skin typically presenting as well-circumscribed erythematous pruritic plaques that can erode, weep, and form crusts 1. Patients are predominantly between 50 and 80 years of age, and the disease is more frequent in females, except in Asian populations 2. The condition is often misdiagnosed as seborrheic dermatitis, lichen sclerosis, Bowen disease, eczema, psoriasis, or a superficial fungal infection, and multiple unsuccessful attempts at dermatologic treatment are common. It is thought to arise from either pluripotent keratinocyte stem cells or epidermal apocrine gland cells, and it leads to a primary adenocarcinoma *in situ* within the epidermis or adnexal structures 3. Less commonly, an underlying adenocarcinoma can extend into the epidermis leading to a secondary form of the disease 3. Malignancies associated with extramammary Paget disease include cancers of the urethra, bladder, prostate, colon, vagina, cervix, or endometrium 4.

Paget cells occasionally exhibit signet ring differentiation because of the presence of intracytoplasmic sialomucin 1. Whereas primary cutaneous or ectodermic extramammary Paget disease expresses the sweat gland immunohistochemical marker pip, the secondary form is usually negative for this marker and instead expresses krt20 5,6.Expression of cea is common to both types. Extramammary Paget disease is sometimes associated with invasion of the dermis or with lymph node involvement, and its metastatic potential is believed to be limited 7,8.

Diagnosis requires a high index of suspicion; the history and physical examination should be directed at identifying an underlying malignancy, particularly if the secondary form of the disease is suspected. Tumour markers, imaging studies, and invasive procedures such as endoscopy or biopsies of other sites should be similarly targeted. Treatment for noninvasive disease is wide local excision, with Mohs surgery achieving improved local recurrence rates 9. Radiation therapy is an acceptable alternative in situations in which surgery might be functionally destructive, or when the disease has recurred after excision 10. No effective chemotherapy regimens for locally advanced or metastatic extramammary Paget disease have been established, and combinations of agents mentioned in case reports include 5fu plus mitomycin C 11, carboplatin plus 5fu and leucovorin 12, low-dose 5fu plus cisplatin 13,14, single-agent docetaxel 15, and mitomycin C plus epirubicin, vincristine, cisplatin, and 5fu 16. response rates cannot be estimated from these studies, but local control and the occasional complete response have been documented.

This case represents a rare presentation of widespread metastases secondary to extramammary Paget disease of the scrotum, with a demonstrated meaningful palliative response to 5fu-based therapy.

## 3. CONFLICT OF INTEREST DISCLOSURE

The contents of this report have not been published or presented elsewhere. All authors meet the criteria for authorship. The authors have no financial interests. No outside support was provided for this research, including funding, equipment, or drugs.
